# Supplementary material for: Phytochemical Profile, Free Radical Scavenging and Anti-Inflammatory Properties of Acalypha Indica Root Extract: Evidence from In Vitro and In Vivo Studies
Source: Molecules. 2021 Oct 15;26(20):6251. doi: 10.3390/molecules26206251 (PMC8537703; doi:10.3390/molecules26206251)
Supplement: Supplementary file 1 [file molecules-26-06251-s001.zip › molecules-1393172-supplementary.pdf]

Phytochemical Profile, Free Radical Scavenging and Anti-inflammatory Properties of *Acalypha Indica* Root Extract: Evidence from In Vitro and In Vivo Studies

Ravi Sahukari[1], Jyothi Punabaka[1], Shanmugam Bhasha[1], Venkata Subbaiah Ganjikunta[1], Shanmugam Kondeti Ramudu[2], Sathyavelu Reddy Kesireddy[1], Weibing Ye[3,*] and Mallikarjuna Korivi[3,*]

Supplementary data

Figure S1. Chromatogram obtained for *A. indica* root methanolic extracts from HRLC-MS/MS analysis.

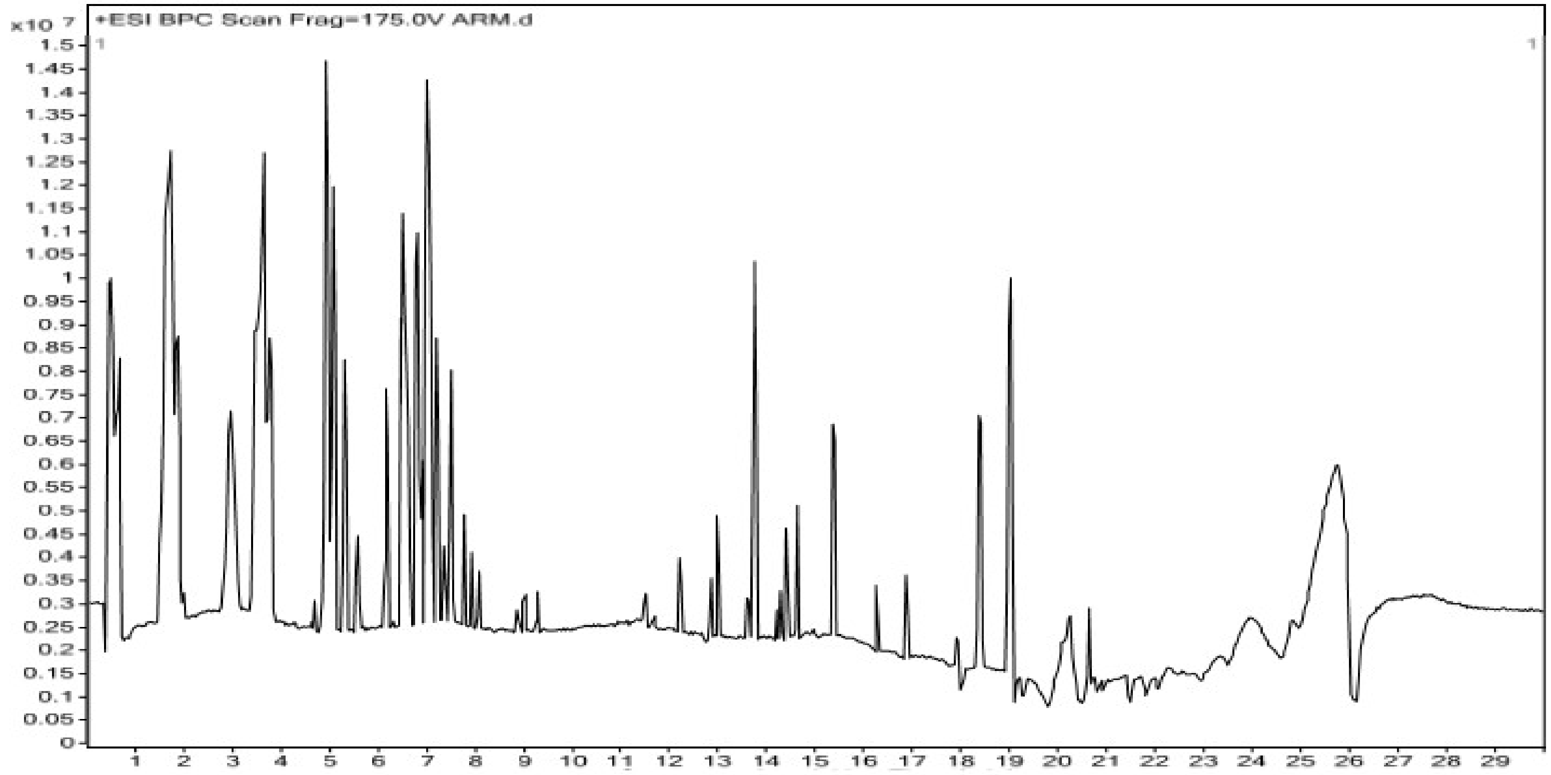

Figure S2. Chromatogram obtained for *A. indica* root methanolic extracts from GC-MS analysis.

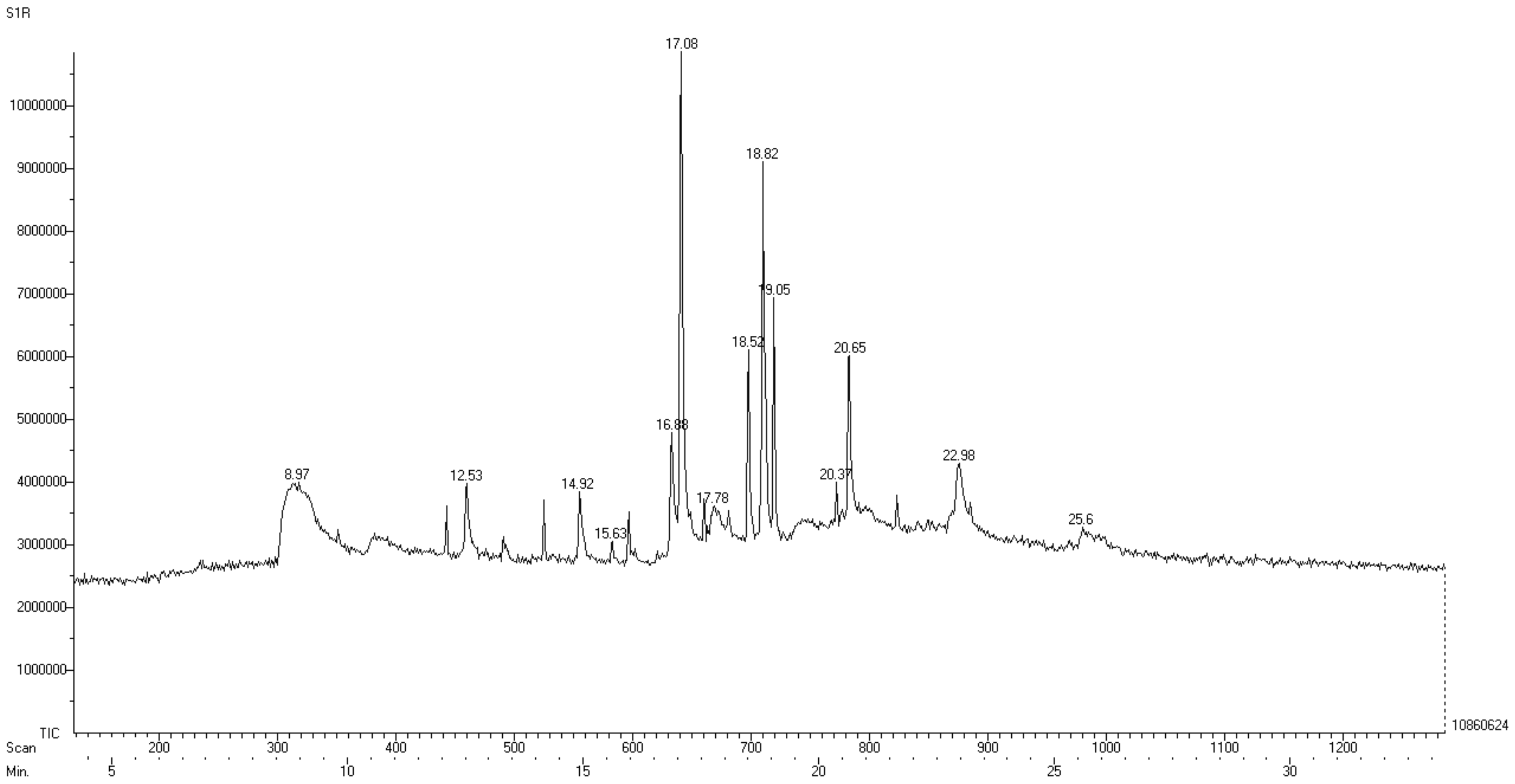

Figure S3. Chemical structures of the constituents identified in *A.indica* root methanolic extracts by HRLC-MS/MS analysis

HRLC-MS/MS

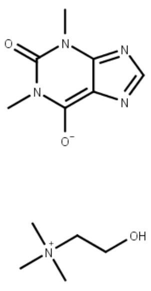

Oxtriphylline

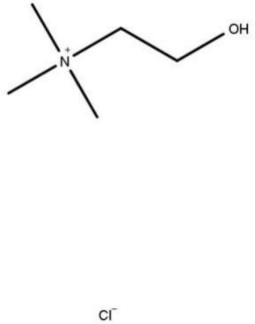

Choline

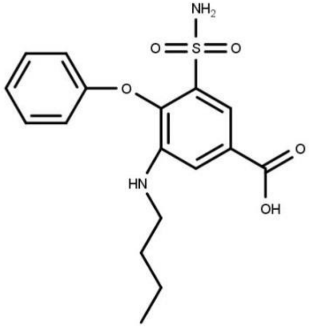

Bumetanide

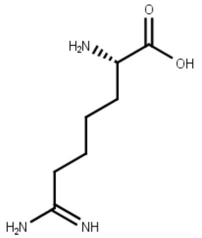

Indospicine

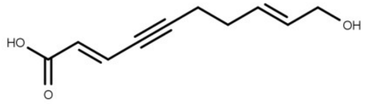

10-hydroxy-2E,8EDecadiene-4,6-diynoic acid

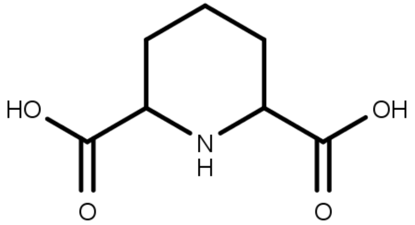

2,6Piperidinedicarboxylic acid

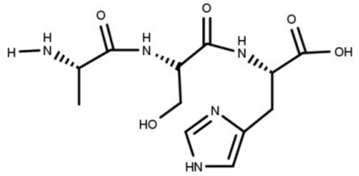

Ala Ser His

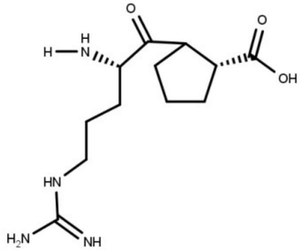

Arg Pro

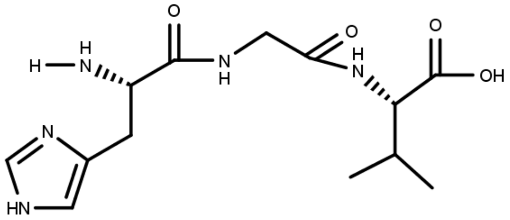

His Gly Val

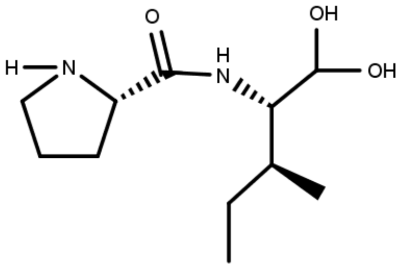

Pro Ile

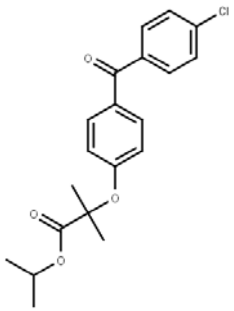

Fenofibrate

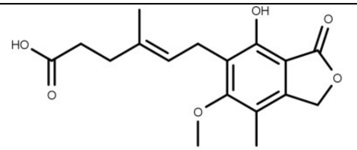

Mycophenolic Acid

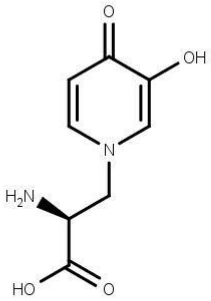<br>**Mimosine**	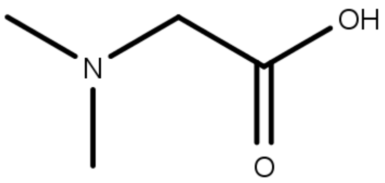<br>Dimethylglycine	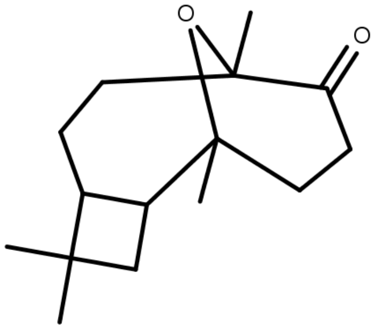<br>3,7-Epoxycaryophyllan-6-One	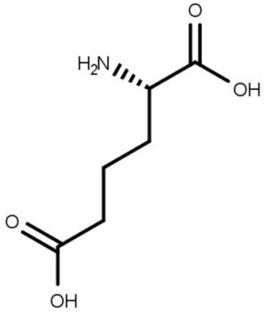<br>**L-2-Aminoadipic acid**
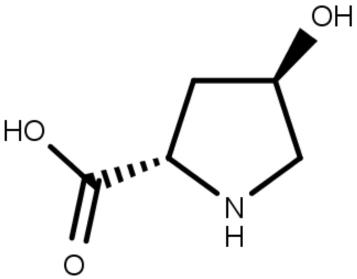<br>**Trans-4-Hydroxy-L-proline**	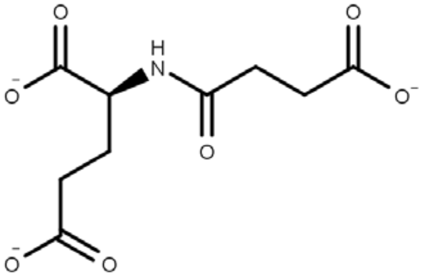<br>N2-Succinylglutamic acid	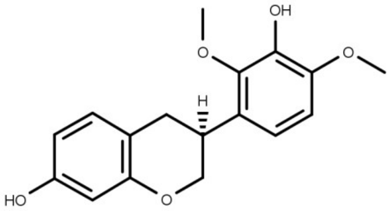<br>Mucronulatol((+/-))	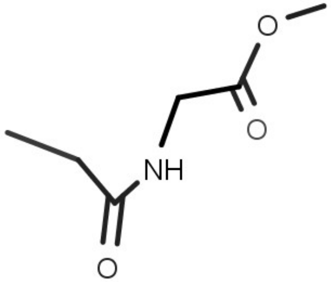<br>Propionylglycine methyl ester
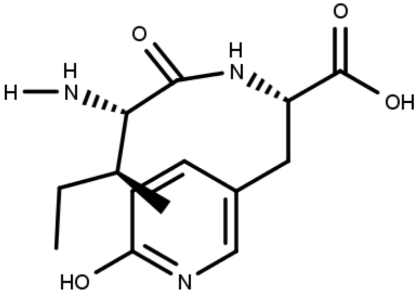<br>Ile Tyr	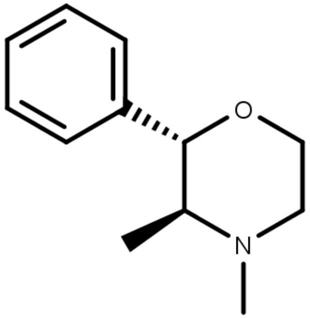<br>Phendimetrazine	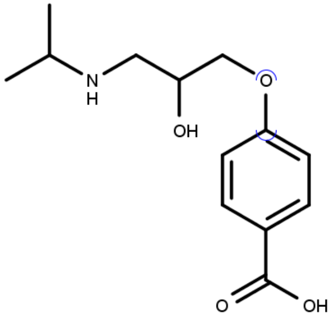<br>4-(2-hydroxy-3isopropylaminopropyl)benzoic scid	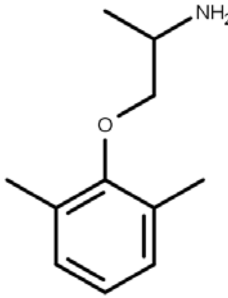<br>**Mexiletine**

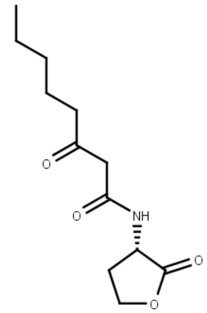<br>**N-(3-oxo-octanoyl)homoserine lactone**	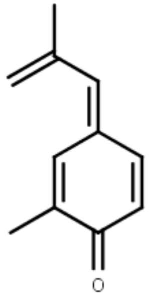<br>Retusoquinone	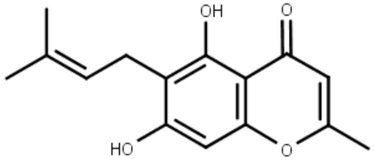<br>Peucenin	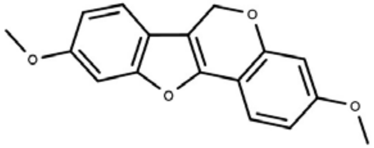<br>Dehydrovariabilin
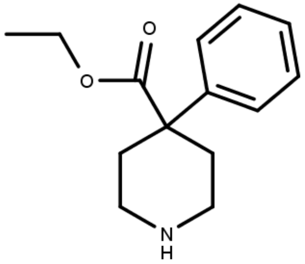<br>Normeperidine	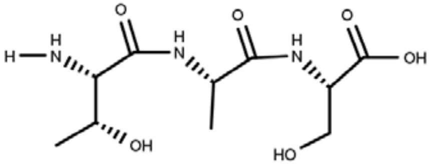<br>Thr Ala Ser	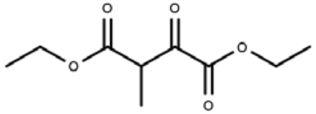<br>**Diethyl Oxalpropionate**	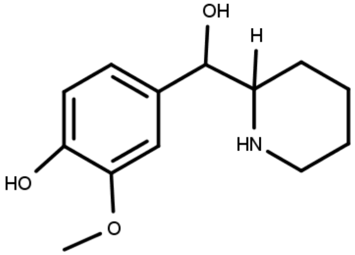<br>3-O-Methylrimiterol
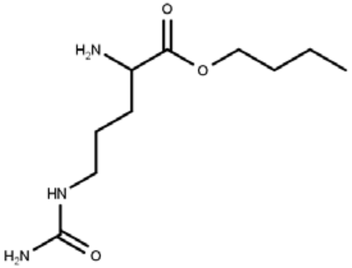<br>Citrulline n-butyl ester	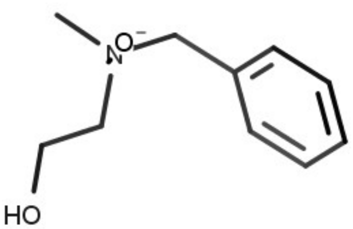<br>2(Benzylmethylamino)Ethanol N-Oxide	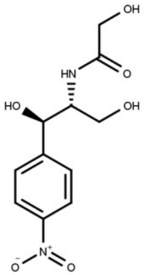<br>**Chloramphenicol alcohol**	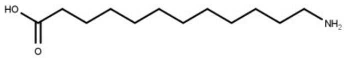<br>**12-aminododecanoic acid**

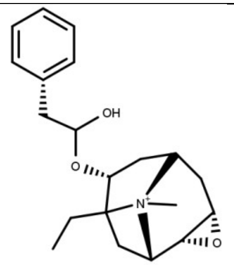 3-Oxa-9azoniatricyclo[3.3.1.02,4]non ane, 9-ethyl-9-methyl-7[(phenylacetyl)oxy]	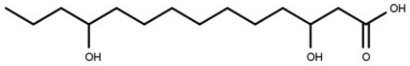 3,11-dihydroxy myristoic acid	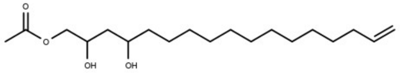 Avocadene Acetate	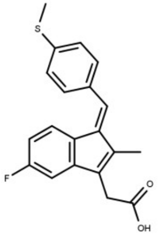 **Sulindac sulfide**
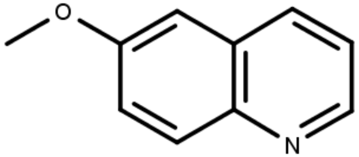 **6-Methoxyquinoline**	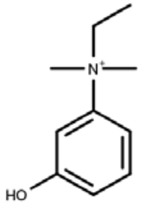 Edrophonium	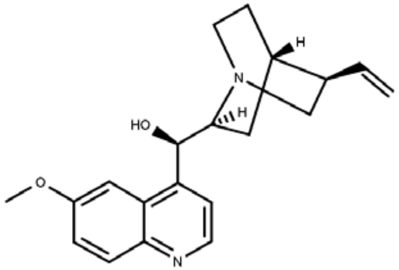 **Quinine**	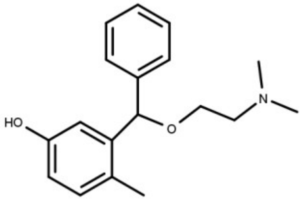 3-[[2(dimethylamino)ethoxy]phenylmethyl]-4-methyl-Phenol
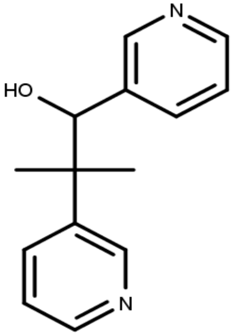 Metyrapol	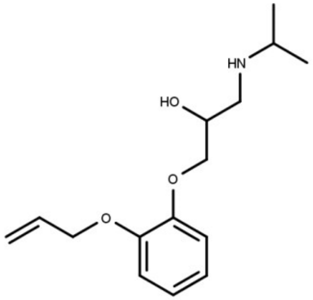 Oxprenolol	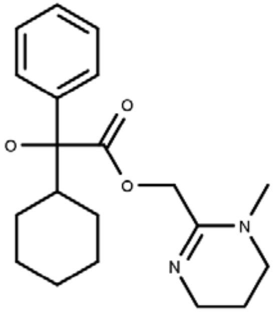 Oxyphencyclimine	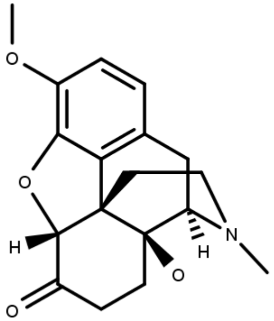 Oxycodone

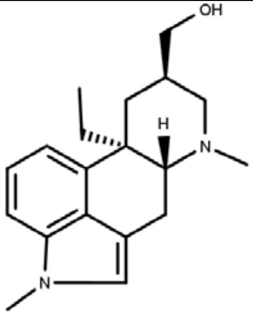

Ergoline-8-methanol, 10-methoxy-1,6-dimethyl

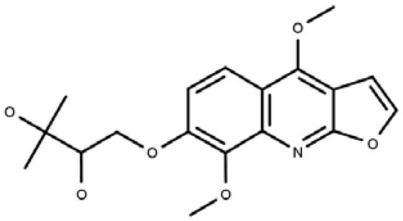

Evoxine

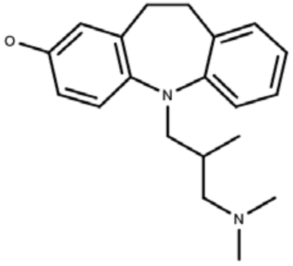

2-Hydroxytrimipramine

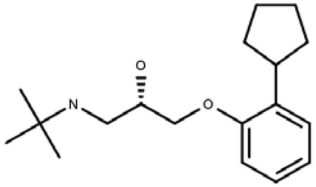

Penbutolol

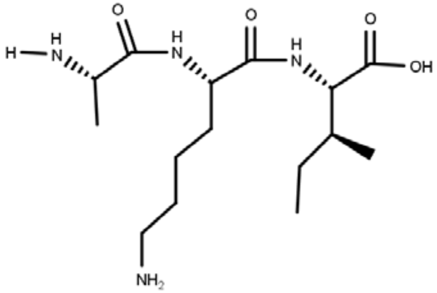

Ala Lys Ile

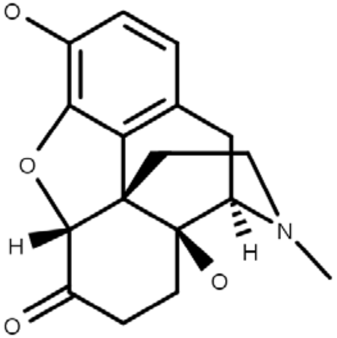

Oxymorphone

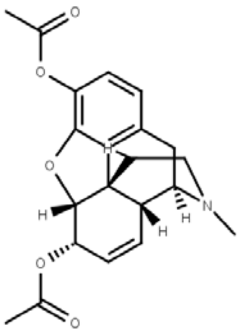

Diamorphine (heroin)

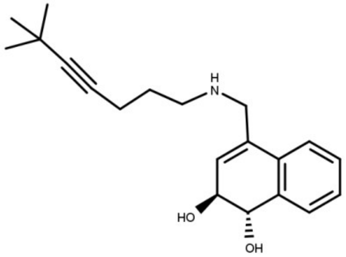

erbinafine metabolite

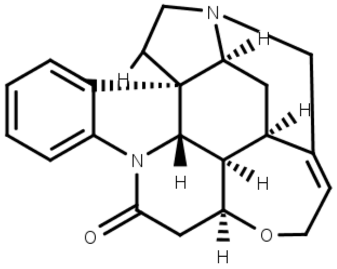

Strychnine

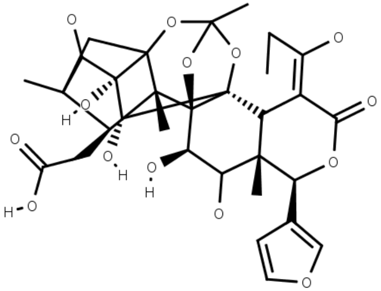

Hydrolysis Product Of Bussein

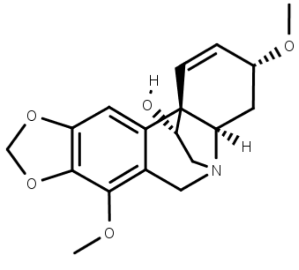

Ambelline

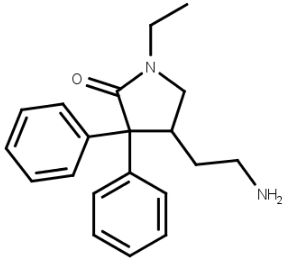

2-Pyrrolidinone, 4-(2aminoethyl)-1-ethyl-3,3diphenyl- (AHR 5904)

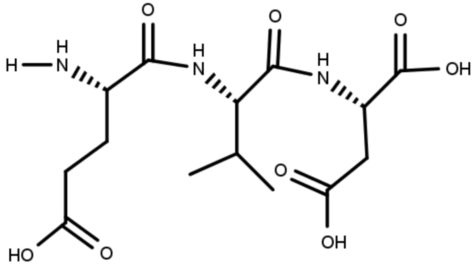 Glu Val Asp	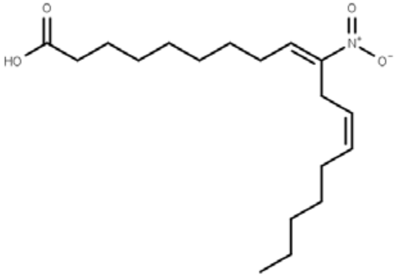 10-nitro,9Z,12Zoctadecadienoic acid	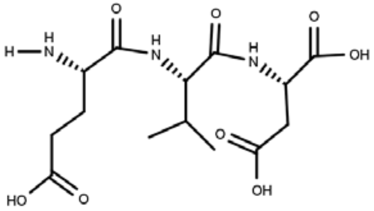 5beta-Chol-2-en-24oic Acid	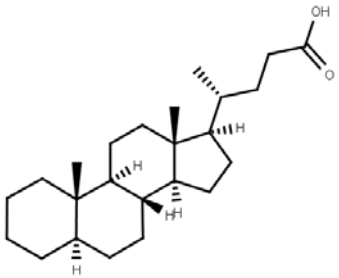 5alpha-Cholan-24oic Acid
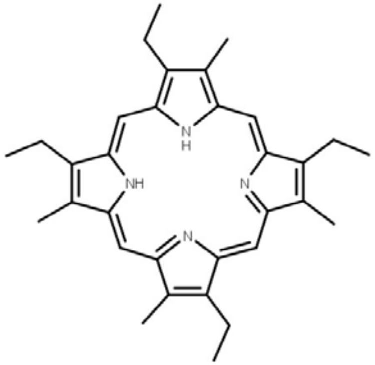 Etioporphyrin III	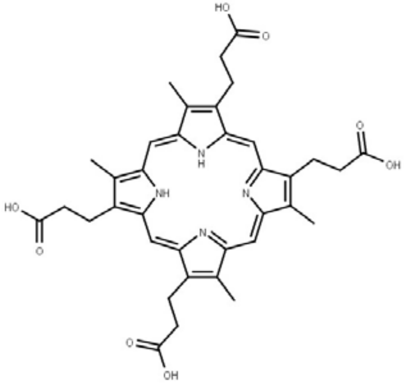 Coproporphyrin II	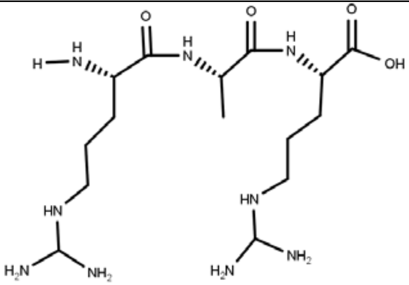 Arg Ala Arg	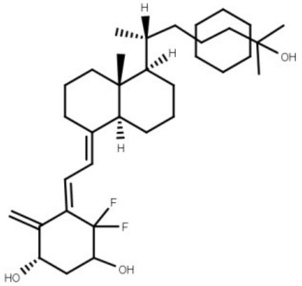 (5Z)-4,4-difluoro1alpha,25-dihydroxyvitamin D3 / (5Z)-4,4-difluoro1alpha,25dihydroxycholecalcifer
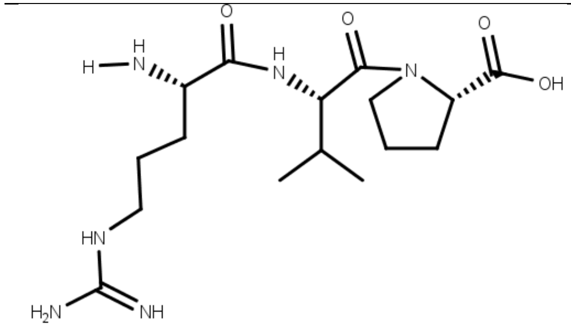 Arg Val Pro	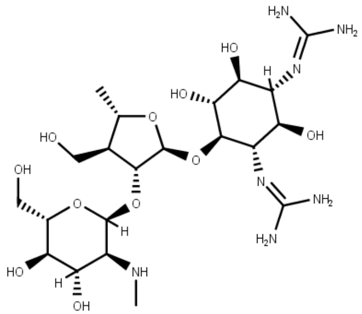 Dihydrodeoxystreptomycin	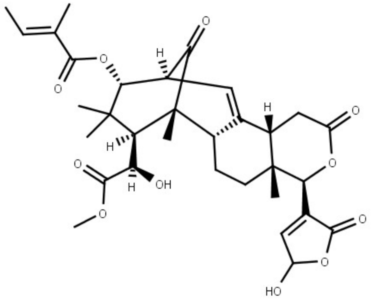 Swietenine	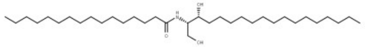 C16 Sphinganine

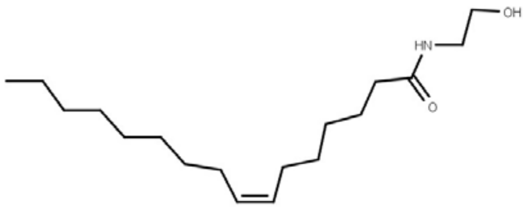 (Z)-N-(2hydroxyethyl)hexadec-7-	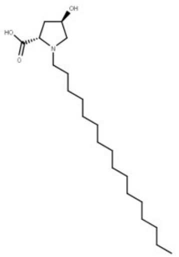 N-Hexadecyl-Lhydroxyproline	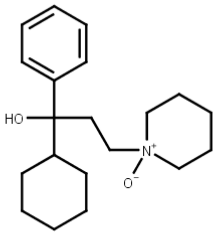 **Trihexyphenidyl N-oxide**	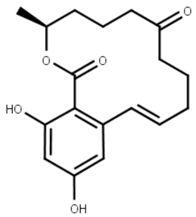 Zearalenone
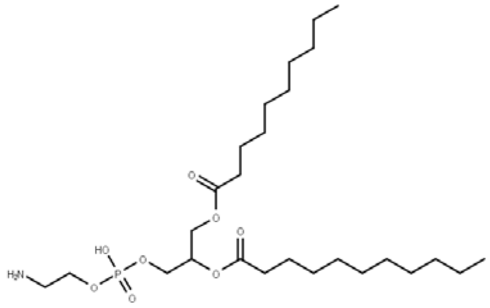 GPEtn(10:0/11:0)[U]	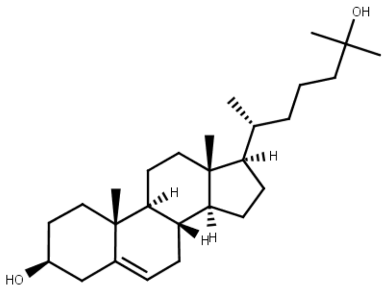 **25-hydroxycholesterol(d3)**	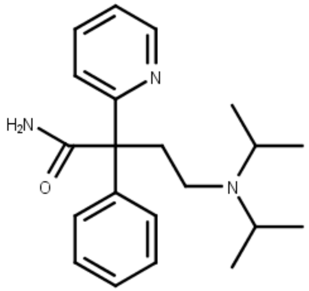 **Disopyramide**	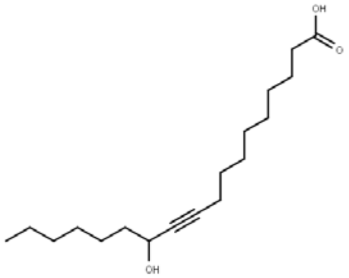 12-hydroxy-10octadecynoic acid
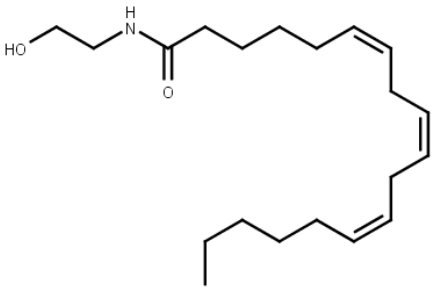 Anandamide (18:3, n-6)	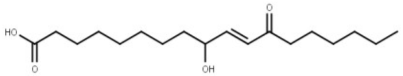 9-hydroxy-12-oxo10-octadecenoic acid	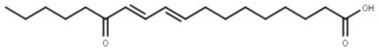 13-OxoODE	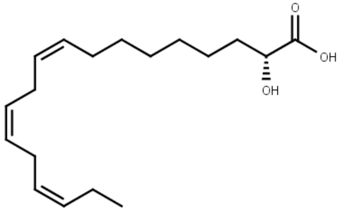 2R-hydroxy9Z,12Z,15Z-octadecatrienoic acid

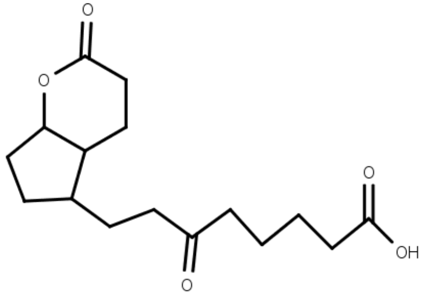 Lactone of PGFMUM	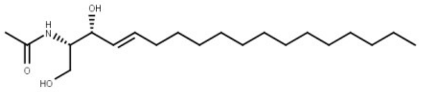 **N Acetylsphingosine**	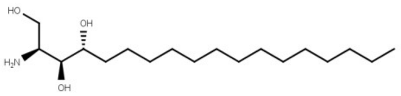 Phytosphingosine	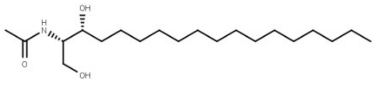 Dihydroceramide C2
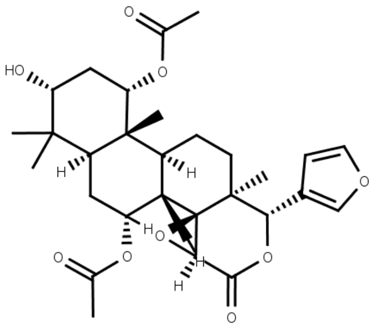 3-Deacetyl Khivorin	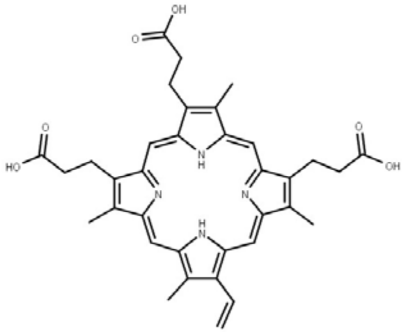 Harderoporphyrin	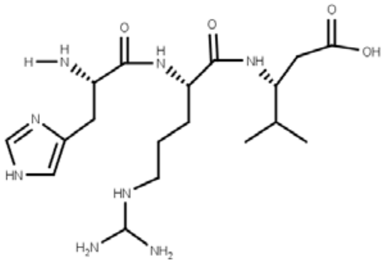 His Arg Val	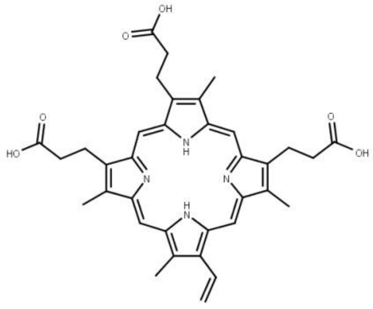 Harderoporphyrinogen
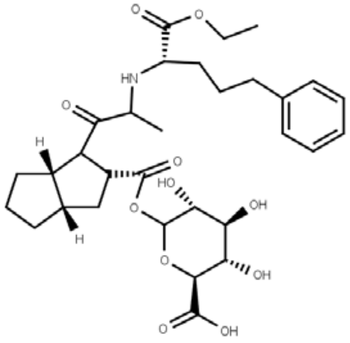 Ramipril glucuronide	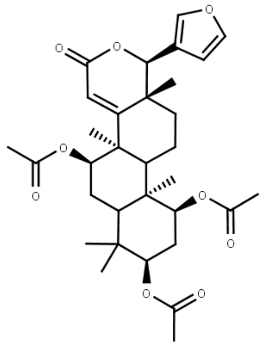 Deoxykhivorin	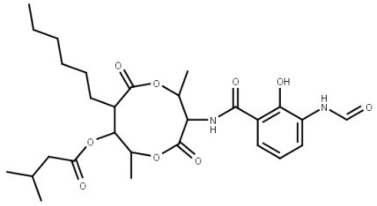 **Antimycin A** (A1 shown)	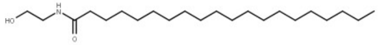 N-(2hydroxyethyl)icosanamide

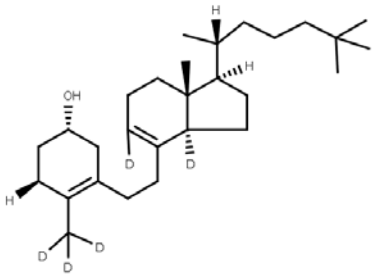<br>9,14,19,19,19pentadeuterio-1alpha,25dihydroxyprevitamin D3 / 9,14,19,19,19-pentadeuterio1alpha,25	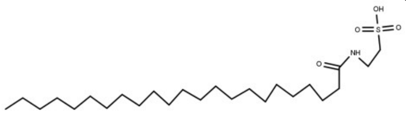<br>2-tricosanamidoethanesulfonic acid	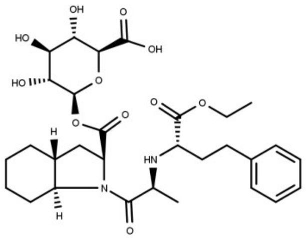<br>Trandolapril glucuronide	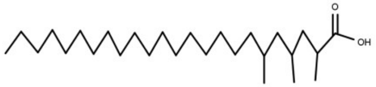<br>2,4,6-trimethyl-2,15tetracosadienoic acid
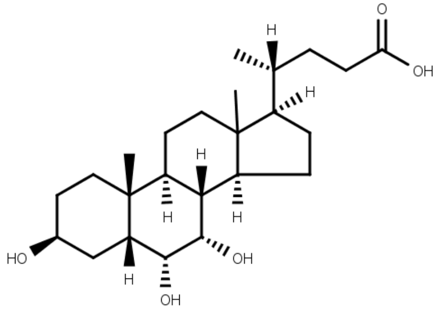<br>3beta,6alpha,7alphaTrihydroxy-5beta-cholan-24oic Acid			

Figure S4. Chemical structures of the constituents ientified in *A. indica* root methanolic extracts by GC-MS analysis.

GC-MS			
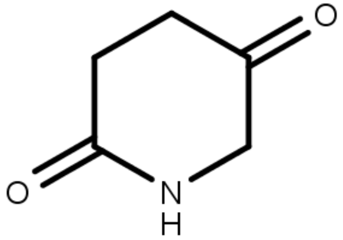<br>Piperidine-2,5-dione	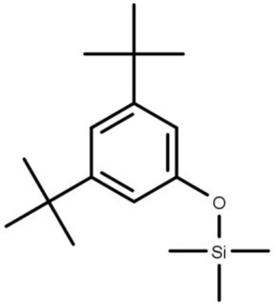<br>Phenol, 2,4-bis(1,1-dimethylethyl)-	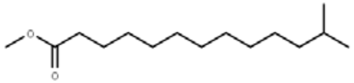<br>Tridecanoic acid,12-methyl-, methyl ester	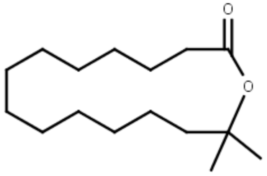<br>1-Oxacyclopentadecan-2-one,15,15-dimethyl
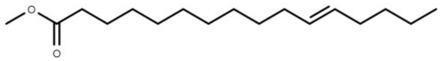<br>11-Hexadecenoic acid, methyl ester	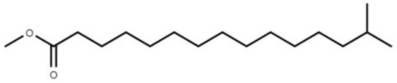<br>Pentadecanoic acid, 14-methyl-, methyl ester	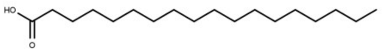<br>Octadecanoic acid	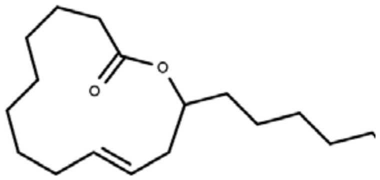<br>13-Hexyloxacyclotridec-10-en-2-one
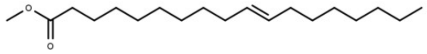<br>10-Octadecenoic acid, methyl ester	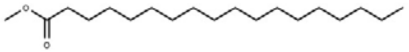<br>Octadecanoic acid, methyl ester	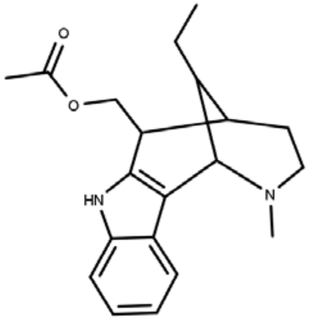<br>Dasycarpidan-1-methanol, acetate (ester)	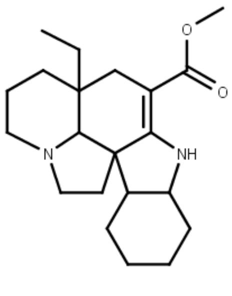<br>Aspidospermidine-3-carboxylic acid, 2,3-didehydro-,methyl ester,[5a,12a,19a]-

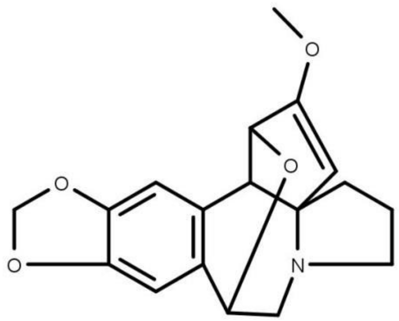 Cephalotaxine,3-deoxy-3,11-epoxy-, (3a,11a)-	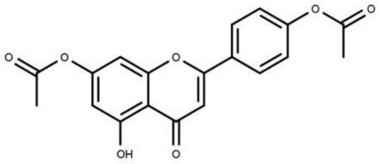 4H-1-Benzopyran-4-one, 7-(acetyloxy)-2-(4-(acetyloxy)phenyl)-5-methoxy-
